# Cell distribution and cytokine levels in induced sputum from healthy subjects and patients with asthma after using different nebulizer techniques

**DOI:** 10.1186/s12890-018-0683-8

**Published:** 2018-07-13

**Authors:** Sinem Koc-Günel, Ralf Schubert, Stefan Zielen, Martin Rosewich

**Affiliations:** 1 Department for Children and Adolescents, Division for Allergology, Pneumology and Cystic Fibrosis, University Hospital Goethe University, Theodor-Stern-Kai 7, 60590 Frankfurt am Main, Germany; 2 Department of Internal Medicine, Division of Pneumology, University Hospital Goethe University, Theodor-Stern-Kai 7, Frankfurt am Main, 60590 Germany

**Keywords:** Induced sputum, Bronchial inflammation, Cell distribution, Smart nebulizer, Ultrasonic nebulizer, Allergic asthma, Cytokines

## Abstract

**Background:**

Sputum induction is an important noninvasive method for analyzing bronchial inflammation in patients with asthma and other respiratory diseases. Most frequently, ultrasonic nebulizers are used for sputum induction, but breath-controlled nebulizers may target the small airways more efficiently. This treatment may produce a cell distribution similar to bronchoalveolar lavage (less neutrophils and more macrophages) and provide deeper insights into the underlying lung pathology. The goal of the study was to compare both types of nebulizer devices and their efficacy in inducing sputum to measure bronchial inflammation, i.e., cell composition and cytokines, in patients with mild allergic asthma and healthy controls.

**Methods:**

The population of this study consisted of 20 healthy control subjects with a median age of 17 years, range: 8–25 years, and 20 patients with a median age of 12 years, range: 8–24 years, presenting with mild, controlled allergic asthma who were not administered an inhaled steroid treatment. We induced sputum in every individual using both devices on two separate days. The sputum weight, the cell composition and cytokine levels were analyzed using a cytometric bead assay (CBA) and by real-time quantitative PCR (qRT-PCR).

**Results:**

We did not observe significant differences in the weight, cell distribution or cytokine levels in the sputum samples induced by both devices. In addition, the Bland-Altman correlation revealed good concordance of the cell distribution. As expected, eosinophils and IL-5 levels were significantly elevated in patients with asthma.

**Conclusions:**

The hypothesis that sputum induction with a breath-controlled “smart” nebulizer is more efficient and different from an ultrasonic nebulizer was not confirmed. The Bland-Altman correlations showed good concordance when comparing the two devices.

**Trial registration:**

NCT01543516 Retrospective registration date: March 5, 2012.

## Background

Sputum induction is a well-known, noninvasive method for analyzing bronchial inflammation in patients with asthma and other respiratory diseases [1, 2]. Standardized induction protocols, including specifications for saline concentrations, the duration and time of induction, and laboratory requirements for sputum processing have been previously described for measuring cell composition, gene expression levels and cytokine patterns [1, 3–6].

Although sputum protocols have been developed to optimize the duration of inhalation and saline concentrations used, few protocols have compared different nebulizers [7, 8]. Davidson et al. compared a vibrating mesh nebulizer with an ultrasonic nebulizer in a previous study but did not detect any differences in sputum cell composition [9].

Several nebulizers are available, and the size of produced respirable particles is important for lung deposition. Conventional nebulizers deliver aerosol particles of approximately 5–10 μg in size, and most droplets are shed in the upper and larger airways. Ultrasonic nebulizers provide smaller droplets of 2–5 μg, and these droplets may be inhaled more easily in the lower airway. For ultrasonic nebulizers, such as the Omron nebulizer, a number of published studies have shown superior efficiency [10]. In addition to particle size, the timing and depth of inhalation are important factors for particle deposition in the lower airways. Recently, so-called smart nebulizers have been developed to more precisely define the breathing maneuvers of patients and target aerosol delivery to specific lung regions [11]. In addition, the use of a smart nebulizer to deliver dornase alpha was recently reported to significantly improve FEF 75% in children with stable cystic fibrosis [12].

Therefore, the technical standardization of sputum induction seems to become more important for more efficiently targeting the small airways and providing deeper insights into lung pathology, comparable to BAL. Based on this information, we investigated whether sputum induction with a smart nebulizer and its technical settings produce a sputum cell distribution similar to BAL. In the present study, we compared sputum weight, cell composition and cytokine levels following treatment with an optimized smart nebulizer and an ultrasonic nebulizer.

## Methods

### Patients

The study included 20 healthy control subjects with a median age of 17 years, range: 8–25 years, and 20 patients with a median age of 12 years, range: 8–24 years, who presented with mild, controlled allergic asthma but were not receiving an inhaled steroid treatment [Table 1]. The patients were recruited from the pediatric outpatient clinic of Goethe University, Frankfurt am Main, Germany, and control subjects were recruited by a public posting. The diagnosis of asthma was based on the Global Initiative for Asthma (GINA).

**Table 1:** Characteristics of patients with allergic asthma and controls

	Controls	Asthmatics
Number	(*n* = 20)	(*n* = 20)
Gender [f/m]	9/11	7/13
Age [age]*	17 (8–25)	13 (8–24)
eNO [ppb]*	15.0 (2.2–35.5)	64.4 (30.4–192.1)
Total IgE [IU/ml]*	72.5 (2–230)	292 (17–1927)
FEV1 [%]*	104.8 (90.1–136.6)	101.5 (51.5–130.5)
VCin [%]*	100.4 (70.7–115.6)	100.5 (69.9–136.2)
FEV1/VCmax [%]*	89.0 (76.25–99.26)	81.9 (51.74–98.15)
RV/TLC [%]*	106.3 (59.58–181.4)	121.2 (54.69–206.3)
MEF 25 [%]*	101.0 (62.7–203.3)	67.5 (20.8–151)

*Data are presented as medians and ranges

The inclusion criteria were: age between 6 and 25 years, informed consent, ability to perform lung function tests, well-controlled allergic asthma, and an exhaled NO (eNO) of eNO > 30 ppb. The exclusion criteria included an acute respiratory illness within four weeks prior to the investigation, other chronic infectious diseases, pregnancy, alcohol/drug/medication abuse and the inability to realize consequences or participation in another study. One patient was excluded due to an asthma exacerbation that was treated with systemic corticosteroids between visits 1 and 2, and another patient did not fulfill the inclusion criteria for allergic asthma. Additionally, one healthy subject did not complete the study because of an infection identified during visit 2.

After providing informed consent, each patient underwent two nonrandomized visits, each of which included a detailed physical examination to evaluate the present status and medical history. Lung function tests, airway reversibility testing and the eNO test were performed. Then, induced sputum was generated as described [13]. Sputum was induced with an ultrasonic nebulizer at visit 1. One week (7 + 5 days) later, at visit 2, lung function and eNO tests were repeated, and sputum was induced with a smart nebulizer.

### Study design

This study was an open, nonblinded explorative study.

### Lung function tests

The lung function tests and reversibility testing were performed using a body plethysmograph (VIASYS Healthcare GmbH, Hoechberg, Germany). The VCmax, FVC, FEV1, FEV1/VC, 25% of the maximum expiratory flow (MEF 25%), RV, and RV/TLC were registered. Lung function tests adhered to the standards of the American Thoracic Society und der European Respiratory Society.

### Exhaled nitric oxide test

Measurements of exhaled NO were conducted using NIOX1 (Aerocrine, Solna, Sweden). NIOX1 measures the eNO in exhaled air, according to the American Thoracic Society guidelines [14]. This chemiluminescence gas analyzer is sensitive to eNO concentrations ranging from 1.5 to 200 ppb and exhibits a deviation from the mean value of + 2.5 ppb at NO 50 ppb or + 5% of the measured value at 150 ppb.

### Description of nebulizers

The ultrasonic device (NE-U17, OMRON® Healthcare Europe, Hoofddorp, Netherlands) uses an ultrasonic frequency of approximately 1.7 MHz to nebulize a volume of up to 4 ml/minute and a particle size of 4.7 μm mass median aerodynamic diameter (MMAD). The airflow and nebulization volumes are adjustable. We used the maximum output of the device for our study, with an airflow velocity of 10 l/s and a nebulization volume of 10 ml/minutes.

The smart nebulizer (AKITA® Jet, Activaero, Gemünden/Wohra, Germany) controls the flow rate and inhalation volume and guides the patient through inhalation [11]. A smart card can be programmed to define the optimal dose of the inhaled particles. In addition, the smart nebulizer provides feedback when the patient, for example, inhales too quickly. The nebulizer creates an individual breathing pattern to optimize the drug delivery with a particle size of 3.8 μm, as measured by the manufacturer.

Nebulization with the ultrasonic nebulizer was tested in a mechanical lung by Activaero GmbH in Gemünden/Wohra, Germany to compare the ultrasonic nebulizer with the smart nebulizer. Measurements showed a delivery of 4 ml NaCl in the lung during an inhalation period of 7 min. The smart nebulizer outputs for our study were adjusted by programming a smart card based on the results, and a peripheral deposition of the aerosol was coded.

### Sputum collection, processing and cell analysis

The patients and controls inhaled saline solutions of 3, 4 and 5% every 7 min, as recently described [13, 15]. During visit 1, subjects inhaled through the ultrasonic nebulizer, and at visit 2, they inhaled through the optimized smart nebulizer.

Shortly after inhalation, the sputum was quantified, and sputum plugs were selected from the samples. Then, 4 × 0.1% (weight/volume) dithiothreitol (DTT) was added, and the samples were processed for 15 min on ice before the subsequent addition of 2 x weight/volume of phosphate-buffered saline (PBS). After centrifuging each sample for 10 min at 790 x *g*, the supernatants were removed by pipette and stored at − 80 °C until further protein analyses. The slides used to analyze cellular differentiation were generated from these samples. Four hundred cells per slide were identified using the Leucodiff 800plus instrument (Instrumentation Laboratory, Bedford, MA, USA), and the percentages of neutrophils, lymphocytes, eosinophils, and macrophages were quantified [13].

### Cytometric bead array (CBA)

The concentrations of four cytokines, IL-5, IL-8, TNF-α and IFN-γ, in sputum samples were determined using the BD™ CBA Flex Set System (BD Biosciences-PharMingen, San Diego, CA, USA). Each BD™ CBA Flex Set contained a one-bead population with distinct fluorescence intensity and both the appropriate phycoerythrin (PE) detection reagent and the standard. The tests were performed according to the manufacturer’s instructions, and samples were tested in duplicate. We added the same concentration of DTT (0.025%) as in the sputum supernatant to the standard curve and the enzyme immunoassay buffer as previously described to analyze the cytokine levels ([13, 15]). The lower detection limits of the cytokines were as follows: IL-8, 1.2 pg/ml; IL-5, 1.1 pg/ml; TNF-α, 0.7 pg/ml and IFN-γ, 1.8 pg/ml.

### RNA extraction

Total RNA was extracted from induced sputum samples using the Qiagen RNeasy Mini Kits (Qiagen, Hamburg, Deutschland), according to the manufacturer’s instructions. All sputum plugs were processed with RNAprotect cell reagent and PBS, according to the manufacturer’s instructions. Before reverse transcription, a DNase treatment was performed using DNase I (Qiagen, Hilden, Germany), as described recently ([15]). The processed RNA samples were supplemented with 9 μL of a master mix of 1 μL of iScript Reverse Transcriptase (Bio-Rad, Hercules, CA, USA), a random hexamer and oligo-dT mix, 4 μL of 10 × iScript RT buffer and 4 μL of nuclease-free water. Then, samples were incubated in a thermocycler at 25 °C for 5 min for an initial incubation step, at 42 °C for 30 min and finally at 85 °C for 5 min.

### Real-time qRT-PCR

Transcripts were quantified using two-step real-time RT-PCR with an Eppendorf Mastercycler RealPlex S detection system (Eppendorf, Hamburg-Eppendorf, Germany) in Greiner 25 μL 96-well reaction plates (Greiner, Germany). The expression of the IL-5, IL-8, TNF-α and IFN-γ mRNAs was normalized to the endogenous control glyceraldehyde-3-phosphate-dehydrogenase (GAPDH), and the relative quantification and calculation of range of confidence was performed using the comparative threshold cycle (2 − ΔΔCt) method (relative gene expression). All amplifications were performed in at least duplicate reactions. The expression data and statistical analysis of the genes involved in immune cells and inflammatory markers were analyzed as previously described [13, 15].

### Data analysis

The data were analyzed with Microsoft Excel (Microsoft Corporation, Redmond, USA), GraphPad Prism 5.0 (GraphPad Software Inc., La Jolla, CA, USA) and BIAS for Windows 11.0 software (Epsilon-Verlag GbR, Hochheim Darmstadt, Germany). The results are presented as medians ± ranges. The differences between the nebulizers were calculated using the Wilcoxon matched-pairs test and the Bland-Altman method. The differences between the patients with asthma and the controls were calculated using the Mann-Whitney test.

## Results

### Sample characteristics

The demographic and clinical characteristics are shown in Table 1. The study population comprised 20 healthy control subjects and 20 patients with mild, controlled allergic asthma who were not receiving steroid treatment. Eighteen patients and 19 healthy subjects completed the study, according to the protocol.

### Sputum

#### Sputum weight

**Fig. 1:**
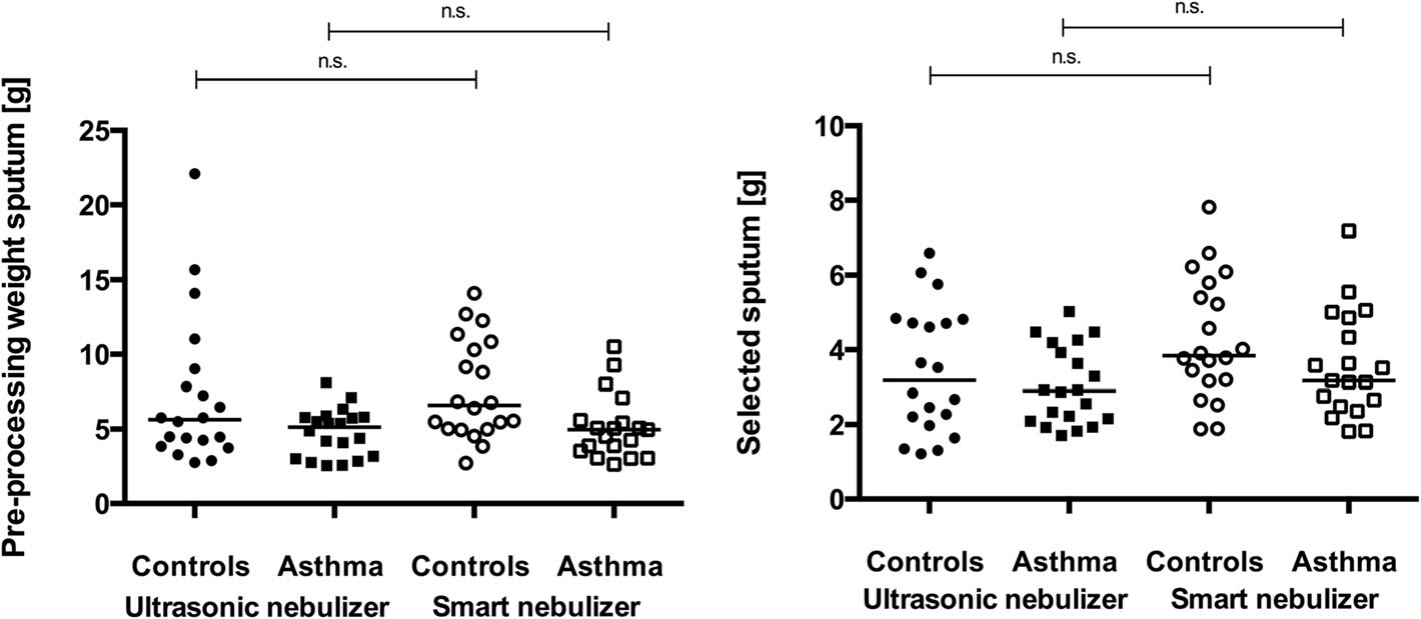
Sputum weight produced using different nebulizers. Data are presented as medians. No differences were observed in preprocessed sputum from the controls (*p* = 0.522) and patients with (*p* = 0.298) [Fig. 1a] or for selected sputum from controls (*p* = 0.143) and patients with asthma (*p* = 0.113) [Fig. 1b] between the ultrasonic and smart nebulizers

The analysis of the sputum weight [g] did not reveal statistically significant differences between the two devices. The median preprocessing sputum weight of the controls was 5.64 [g] (2.77–22.09) at V1 and 6.60 [g] (2.72–14.10) at V2. For patients with asthma, the median preprocessing sputum weight was 5.14 [g] (2.55–8.09) at V1 and 4.97 [g] (2.64–10.52) at V2. The median weight of the induced sputum in the group of controls was 3.19 [g] (1.22–6.6) at V1 and 3.85 [g] (1.89–7.82) at V2. In patients with asthma, the median weight was 2.90 [g] (1.71–5.03) at V1 and 3.18 [g] (1.82–7.19) at V2 [Fig. 1a and b].

#### Sputum cell count

**Fig. 2:**
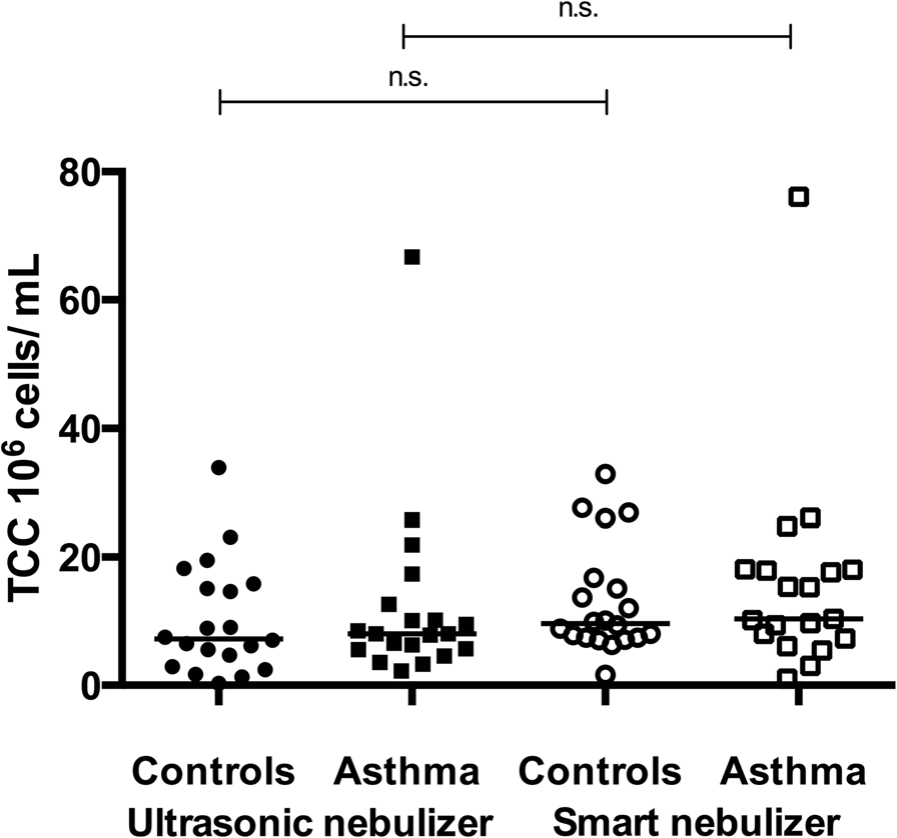
Total cell counts obtained using different nebulizers. Data are presented as medians. In the controls, *p* = 0.325 when comparing ultrasonic and smart nebulizers. In patients with asthma, *p* = 0.275

Total cell counts [106 cells/ml] were compared between the controls and patients with asthma and between the nebulizers. A difference in the total cell counts in induced sputum was not observed between the devices in either group (controls: *p* = 0.41; patients with asthma: *p* = 0.33) [Fig. 2].

#### Sputum cell composition

**Fig. 3:**
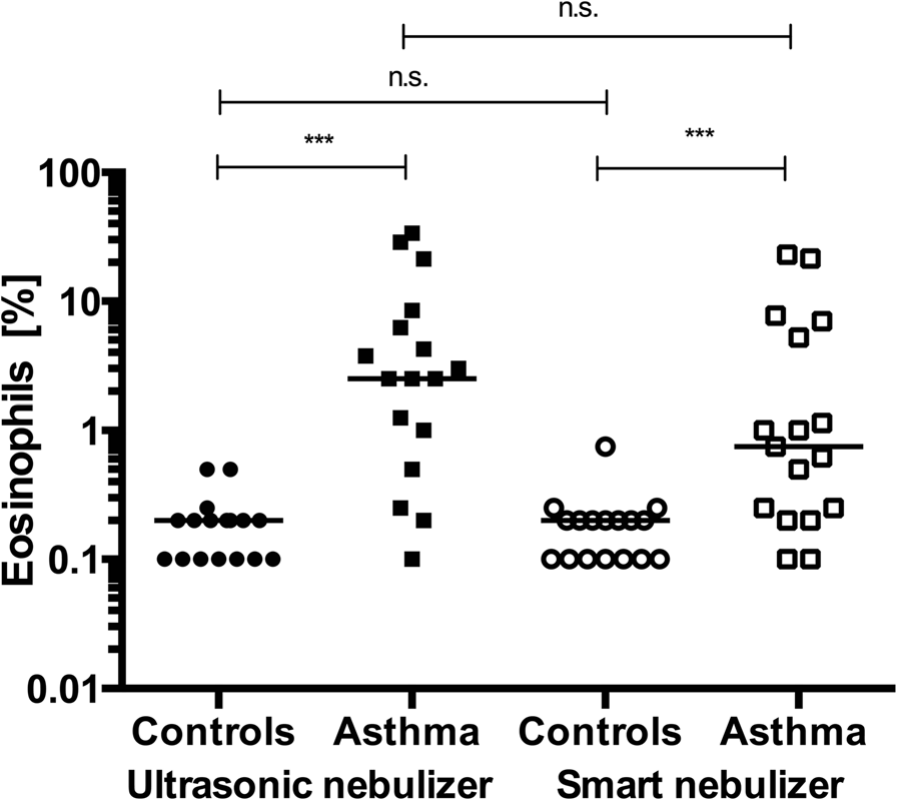
Percentage of eosinophils obtained after the use of different nebulizers. Data are presented as medians. Zero values were increased to 0, 1/0, or 2 for visualization. In the controls, *p* = 0.670 when comparing ultrasonic and smart nebulizers. In the patients with asthma, *p* = 0.816. The percentage of eosinophils was significantly elevated in patients with asthma compared to that in controls

Each cell subtype was compared between the sputum collected with the ultrasonic and smart nebulizers. A comparison of the percentage of macrophages after induction with the ultrasonic or the smart nebulizer did not show significant differences in either group (controls: *p* = 0.605; patients with asthma: *p* = 0.737). Additionally, a significant difference in the percentage of neutrophils after the use of the different devices was not observed in either group (controls: *p* = 0.670; patients with asthma: *p* = 0.816). The percentages of eosinophils did not differ between sputum collected with the different devices (controls: *p* = 0.344; patients with asthma: *p* = 0.224), but the percentage of eosinophils differed significantly between patients with asthma and controls based on the inclusion criteria (*p* < 0.0001 at V1; *p* = 0.0003 at V2). The results are shown in Fig. 3.

### Estimation of cytokine levels

**Fig. 4:**
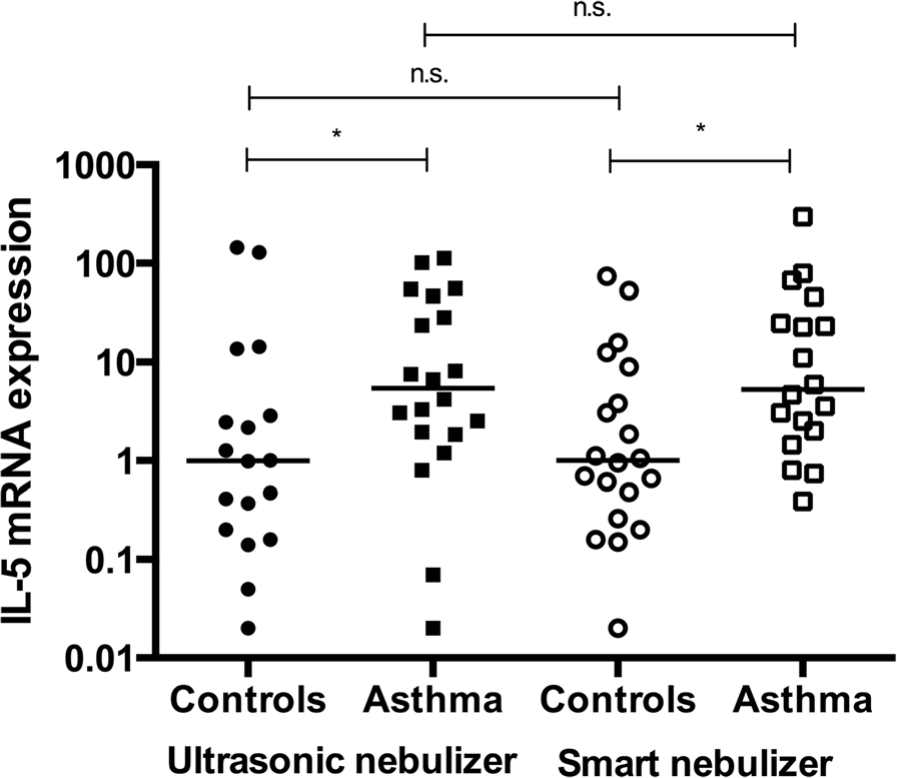
IL-5 mRNA expression measured after the use of different nebulizers. Data are presented as medians. In the controls, *p* = 0.3927 when comparing the Omron and Akita nebulizers. In the patients with asthma, *p* = 0.4307. qRT-PCR revealed a significant elevation in IL-5 levels in patients with asthma compared with levels in controls

The cytokine levels measured by qRT-PCR and CBA were compared between the devices. No significant differences were observed for levels of the cytokine proteins and mRNAs between the sputum collected with the two devices. As expected, qRT-PCR revealed that patients with asthma had significantly higher levels of IL-5 than controls (*p* = 0.0360 at V1 and *p* = 0.0115 at V2) [Fig. 4]. IL-8 and IFN-γ expression (IL-8 in controls *p* = 0.420 vs. IL-8 in patients with asthma *p* = 0.7439 and IFN-γ in controls *p* = 0.695 vs. IFN-γ in patients with asthma *p* = 0.327) were not different between the patient groups. In addition, no differences in cytokine levels measured using CBA were identified between the two nebulizers.

### Bland-Altman correlation

The correlation between both devices was analyzed using the Bland-Altman method, which compares differences in two methods using their means. We compared the cell counts obtained after the use of both devices. A small bias of − 0.2441 and a SD of 9.614 were found for the macrophage population, and a bias of − 0.5075 and a SD of 8.016 were identified for the neutrophils. The results are shown in Table 2.

**Table 2:** Bland-Altman analysis of both nebulizers

	Macrophages	Neutrophils	All cell types
Bias	−0.2441	−0.5075	− 0.001625
SD of bias	9.614	8.016	6.153
95% limits of agreement			
From	−19.09	−16.22	−12.06
To	18.60	15.20	12.06

## Discussion

Because sputum induction is a noninvasive method for evaluating bronchial inflammation, particularly for diagnostic research purposes, the induction methods, including devices and their technical settings, must be standardized [16]. For this purpose, the sputum weight, cell composition and cytokine levels were compared in patients using the ultrasonic and smart nebulizers. Interestingly, we did not observe differences between the different sputum induction devices.

The total deposition of aerosols in the lungs depends on the particle size, breathing pattern, and lung volume [17]. In addition, factors such as saline concentrations and the duration of inhalation influence both the cell distribution and the quality of sputum [18–20]. Several studies have reported an increasing percentage of macrophages for longer time intervals of sputum induction, reflecting a cell distribution consistent with the peripheral airways [18]. According to the study by Gershman et al., shorter induction periods produce a higher percentage of neutrophils, which represent the large airways, but longer induction periods result in an increasing percentage of macrophages, which are most likely derived from the small airways [5]. More recently, smart nebulizers have been able to guide patients to inhale slowly and deeply to more effectively deposit inhaled particles in the small airways [12, 21, 22].

However, the hypothesis that sputum induction with a smart nebulizer, here, the Akita Jet, and its technical settings produce a sputum cell distribution similar to BAL was not confirmed in our study. In addition, significant differences in any of the investigated parameters, including gene expression levels and cytokine patterns, were not observed between the two devices. One criticism is that the nonrandomized design of our study may induce a small learning effect in favor for the Akita Jet results. However, when we considered the difference in handling of both nebulizers, the learning effect was neglected. The Bland-Altman correlations showed good concordance when comparing the two devices. This finding is contradictory to previous studies showing that smart nebulizers are more efficient drug delivery devices that deposit larger amounts of the inhaled dose in the small airways [12, 21, 23, 24].

One possible explanation for this discrepancy is related to the saline particles. Hygroscopic particles such as NaCl tend to increase in size when passing through the lung due to humidity, thus limiting the diameter for the minimum deposition in the peripheral lung [25]. Additionally, the initial diameter of the particle sizes creates a difference in deposition [25]. Although the use of small particle sizes (0.1 μm) shows a similar distribution, regardless of the presence of nonhygroscopic or hygroscopic particles, because their primary deposition mechanism is diffusion, the use of larger sizes (1 μm) alters the deposition due to particle growth, thereby altering the deposition pattern [25]. Further investigations are necessary to evaluate the effect of NaCl particle sizes on sputum induction.

Conversely, the inhalation of an aerosol, regardless of whether it is deposited in central or peripheral airways, must not lead to a higher expectoration of sputum and inflammatory cells. Because the underlying pathological mechanism of asthma involves hyperplasia of the smooth muscle, the expectoration of peripheral sputum is likely limited due to decreases in the diameter of the lumen, which might retain secretions. Indeed, Pavia et al. have identified a positive correlation between patients’ FEV1 and the depth of deposition [26–28]. In addition, cough is less effective in the small airways of the lung, as shown in the study by Alexis et al. [29]. The authors showed that induced sputum samples are predominantly derived from the central airways, and little or no clearance is associated with sputum induction when a radio aerosol was targeted to the alveolar region.

Finally, breathing patterns have been shown to influence lung deposition in several studies [25, 27, 30]. Although the subjects were instructed to avoid shallow breathing and to take deep breaths in each session, the breathing patterns were not identical for both nebulizers. During inhalation with the smart nebulizer, the subjects made small pauses due to the previously programmed breathing pattern, but the subjects inhaled continuously when using the ultrasonic nebulizer. A tidal breathing pattern that precludes any deep breaths during inhalation may enhance the degree of induced bronchoconstriction [31]. In contrast, the smart nebulizer guided subjects to pause and take the mouth piece out to open up the lungs using a deeper inhalation strategy. Surprisingly, although breathing patterns were optimized by the smart nebulizer, the expectorated sputum did not differ from that samples produced after the use of the ultrasonic nebulizer.

## Conclusions

In conclusion, we did not detect differences in sputum induction between the ultrasonic nebulizer and smart nebulizer. The sputum weight, the cell composition and cytokine levels showed good concordance when comparing the two devices. In particular, for academic purposes, additional investigations aiming to standardize the technical settings of sputum induction should be performed in the future.

## Abbreviations

BAL: Bronchoalveolar lavage
BIAS: Bias function
CBA: Cytometric bead array
DNA: Deoxyribonucleic acid
DTT: Dithiothreitol
ECP: Eosinophil cationic protein
EnO: Exhaled nitric oxide
FEV1: Forced expiratory volume in 1 s
FEV1/VC: Tiffeneau index
FVC: Forced vital capacity (FVC)
IFN- γ: Interferon gamma
IL-5: Interleukin-5
IL-8: Interleukin-8
MEF25: 25% of the maximal expiratory flow
MMAD: Mass median aerodynamic diameter
mRNA: Messenger ribonucleic acid
NaCl: Sodium chloride
PBS: Phosphate-buffered saline
PCR: Polymerase chain reaction
RV: Residual volume
RV/TLC: Functional residual volume
SD: Standard deviation
V1: Visit 1
V2: Visit 2
VC: Vital capacity
